# Crop Rotation with Marigold Promotes Soil Bacterial Structure to Assist in Mitigating Clubroot Incidence in Chinese Cabbage

**DOI:** 10.3390/plants11172295

**Published:** 2022-09-02

**Authors:** Jinhao Zhang, Waqar Ahmed, Xinghai Zhou, Bo Yao, Zulei He, Yue Qiu, Fangjun Wei, Yilu He, Lanfang Wei, Guanghai Ji

**Affiliations:** 1 State Key Laboratory for Conservation and Utilization of Bio-Resources in Yunnan, Yunnan Agricultural University, Kunming 650201, China; 2 Key Laboratory of Agro-Biodiversity and Pest Management of Ministry of Education, Yunnan Agricultural University, Kunming 650201, China; 3 Agricultural Foundation Experiment Teaching Center, Yunnan Agricultural University, Kunming 650201, China

**Keywords:** *Plasmodiophora brassicae*, bacterial community, crop rotation, resting spores, marigold

## Abstract

Clubroot caused by *Plasmodiophora brassicae* is an economically important soilborne disease of Chinese cabbage worldwide. Integrated biological control through crop rotation is considered a good disease management approach to suppress the incidence of soilborne diseases. In this study, we evaluated the effect of a marigold plant (root exudates, crude extract, and powder) on the germination and death of resting spores of *P. brassicae* in vitro assays. Additionally, we also performed 16S high throughput sequencing, to investigate the impact of marigold–Chinese cabbage crop rotation on soil bacterial community composition, to manage this devastating pathogen. This study revealed that the marigold root exudates, crude extract, and powder significantly promoted the germination and death of *P. brassicae* resting spores. Under field conditions, marigold–Chinese cabbage crop rotation with an empty period of at least 15 days enhanced the germination of *P. brassicae* resting spores, shifted the rhizosphere bacterial community composition, and suppressed the incidence of clubroot by up to 63.35%. Proteobacteria, Acidobacteria, Bacteroidetes, Actinobacteria, and Verrucomicrobia were the most dominant phyla and were present at high relative levels in the rhizosphere soil of Chinese cabbage. We concluded that crop rotation of Chinese cabbage with marigold can significantly reduce the incidence of clubroot disease in the next crop. To our knowledge, this is the first comprehensive study on the prevention and control of clubroot disease in Chinese cabbage through crop rotation with marigold.

## 1. Introduction

Clubroot caused by the soilborne obligate parasite *Plasmodiophora brassicae* is a serious threat to Chinese cabbage (*Brassica rapa* subsp. *Pekinensis*) and plants belonging to the Brassicaceae family, affecting production worldwide, including in China [1]. The disease occurs in more than 60 countries and results in a 10 to 15% reduction in yield on a global scale [2]. Incidence of clubroot disease has been reported in all major rapeseed-producing areas of China, among which the regions of Chongqing, Hubei, Sichuan, and Yunnan are badly affected by this pathogen [3]. In China, the average yield losses are recorded between 20 to 30%, and the disease is characterized as stunting plant growth with yellowing of leaves and massive galls or club formation on the roots [1,4]. The pathogen survives in the soil as resting spores for up to 20 years, making it difficult to control the clubroot disease completely [5].

Several alternative means have been proposed to control plant diseases by inducing resistance in the plants, by the application of different protein hydrolysates [5] and salt solution treatment [6,7]. The important methods to control clubroot disease are liming [8]; agrochemicals, such as cyazofamid, chlorothalonil, carbendazol, and fluazinam [3,9]; resistant cultivars [10]; and crop rotation [11]. Previous studies have reported that using a monocropping system is a common agricultural practice worldwide and is associated with the acceleration of soilborne diseases [12,13]. It has been proven that the plant rhizosphere acts as a hotspot ecological environment for plant–microorganism interaction [14]. Rhizosphere microbes play a significant role in maintaining soil health, plant growth and health, and disease suppression [15,16]. Maintaining the diversity of plant species in an ecosystem using crop rotation and intercropping protects plants from biotic stresses [17], by improving the diversity and structure of rhizosphere microorganisms [18]. Similarly, biocontrol, through potent antagonistic microbes, protects plants from soilborne pathogens by induction of host resistance, niche exclusion, and direct antagonism [19].

Marigold (*Tagetes erecta* L.) is an economically important ornamental plant, well-known for its medicinal and antimicrobial properties [20]. There are many reports on the successful use of marigolds to control crop diseases [20,21]. Marigold produces allelopathy compounds with antimicrobial activity and can antagonize 14 genera of plant-parasitic nematodes [22]. Marigold essential oil showed strong antibacterial and fungal activity against many pathogenic bacteria and fungi [23]. Previous studies have reported that marigold crop rotation, intercropping, and cover cropping could effectively suppress the infestation of nematodes in eggplant, okra, tomato, and soybean [22,24,25]. Intercropping with marigold promotes soil health and microbial structure, to mitigate tobacco bacterial wilt disease [21] and early blight disease in tomato [20]. Integrated treatment of marigold and *B. amyloliquefaciens* ZM9 significantly suppressed the incidence of tobacco bacterial wilt disease compared with a single application of marigold and *B. amyloliquefaciens* ZM9 [26]. It has been reported that marigold root-exudates are lethal to root-knot nematodes (RKNs) and marigold acts as a trap plant for RKNs when planted near to the host plants [27]. The organic extracts of macroalgae exhibited antagonistic activity against *Fusarium oxysporum*, the causative agent of *Fusarium* wilt disease of tomato [28]. Similarly, Canada milkvetch extract significantly suppressed the incidence of *Verticillium* wilt in potato, with a relative control effect of about 55–84% [29]. Thus, we assumed that integrated disease management (IDM) approaches could effectively control the incidence of soilborne diseases.

Traditionally, clubroot in the Brassicaceae family has been managed by rotation with gramineous (maize, rice) and leguminous (clover, soybean) crops [30], and there have been no reports on the use of marigold crop rotation to mitigate clubroot in cruciferous crops. In this study, we investigated the effect of marigold powder, crude extract, and root exudates on the germination and death of *P. brassicae* resting spores in vitro, as well as investigating marigold crop rotation on the incidence of clubroot of Chinese cabbage in greenhouse and field experiments. This study aimed to develop effective IDM approaches for green and sustainable agriculture, to mitigate the incidence of clubroot in Chinese cabbage.

## 2. Results

### 2.1. Marigold Roots Enhance the Germination of P. brassicae Resting Spores

**Figure 1:**
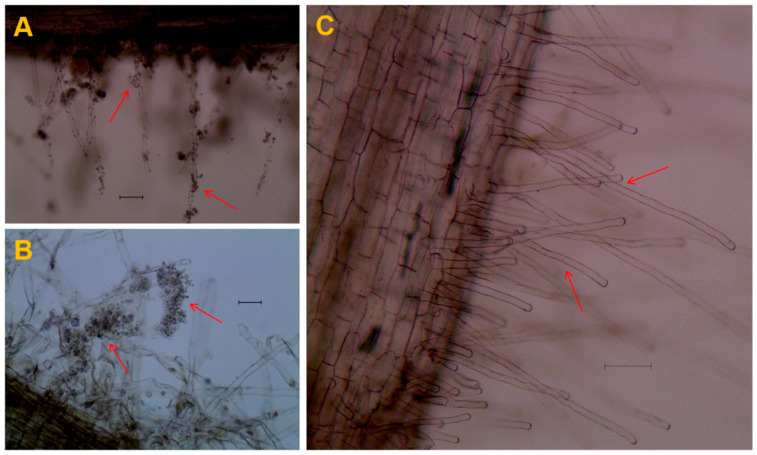
Confocal microscopy micrographs of marigold roots inoculated with *Plasmodiophora brassicae* resting spores. (**A**,**B**); Inoculated with 100 μL/tube of *P. brassicae* spore suspension (1 × 10^7^ spores/mL) and (**C**); inoculated with sdH_2_O 100 μL/tube as control. Red arrows show the germination of *P. brassicae* spores after 24 h post-inoculation (**A**), 7 days post-inoculation (**B**), and with no spores (**C**). Bars represent 50 μM.

Marigold seedlings were grown hydroponically in the dark in test tubes and treated with 100 μL/tube of *P. brassicae* spore suspension (1 × 10^7^ spores/mL) and sdH_2_O as control (CK). Root hairs were collected after 24 h and 7 days post-inoculation with *P. brassicae* and visualized under a confocal microscope (Figure 1). It was found that germination of *P. brassicae* resting spores started after contact with marigold roots. After 24 h of post-inoculation with *P. brassicae*, the spores started germinating and were observed on the surface of root hairs (Figure 1A). Whereas, after 7 days of post-inoculation, zoosporangium appeared, spores clumped together, and swimming was observed (Figure 1B). However, no spores were observed on marigold roots treated with sdH_2_O (Figure 1C).

### 2.2. Marigold Root Exudates, Crude Extract, and Powder Influence the Germination and Death of P. brassicae Resting Spores

**Figure 2:**
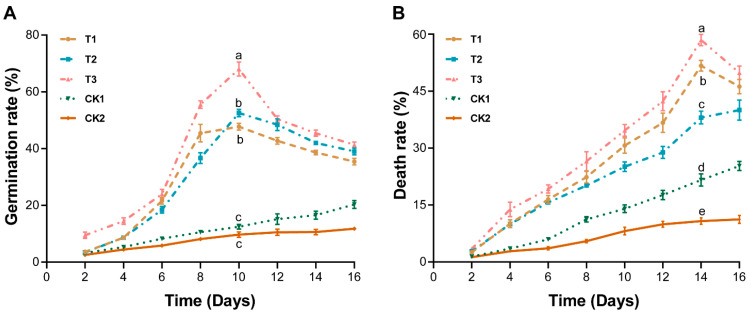
Effect of marigold root exudates, crude extract, and powder on the germination and death of *Plasmodiophora brassicae* resting spores after specific days of treatment. Germination (**A**) and death (**B**) rate of *P. brassicae* resting spore under different treatments. Here; Marigold root exudates (T1), crude extract (T2), powder (T3), methanol (CK1), and sdH_2_O (CK2). Different lowercase letters on the error bars show significant differences among treatments according to Duncan’s multiple range test at *p <* 0.05.

The effect of marigold root exudates, crude extract, and powder on the death and germination of *P. brassicae* resting spores was observed after 2 days post-treatment, with an interval of 2 days to 16 days (Figure 2). It was found that the marigold root exudates (T1), crude extract (T2), and powder (T3) significantly promoted the germination (Figure 2A) and death (Figure 2B) of *P. brassicae* resting spores compared with methanol (CK1) and sdH_2_O (CK2). A similar trend was observed for the germination and death of *P. brassicae* resting spores under all treatments, which first increased and later decreased with an increase in incubation time. The germination and death rates of *P. brassicae* resting spores recorded a maximum after 10 days (Figure 2A) and 14 days (Figure 2B) post-treatment, respectively, under all treatments. In contrast, the treatment (T3) application of marigold powder was found to be best, as it significantly enhanced the germination and death of *P. brassicae* resting spores. Thus, based on these results, the marigold powder was found to be best and selected for the subsequent greenhouse assay.

### 2.3. Effect of Marigold Powder on the Incidence of Clubroot in Chinese Cabbage

A pot experiment was conducted in a greenhouse under controlled environmental conditions, using Chinese cabbage seedlings treated with marigold powder and *P. brassicae* spore suspension (1 × 10^7^ spores/mL). The results demonstrated that the combined application of marigold powder (T1) suppressed the incidence of clubroot in Chinese cabbage, having a control effect of about 21.36% compared to spores treated with marigold powder with control (CK) (Table 1). Whereas, when *P. brassicae* was grown in test tubes 15 days before the seedling, a control effect was achieved of up to 47.41% (Table 1).

### 2.4. Marigold-Chinese Cabbage Crop Rotation Suppresses the Incidence of Clubroot in Chinese Cabbage under Greenhouse and Field Conditions

**Figure 3:**
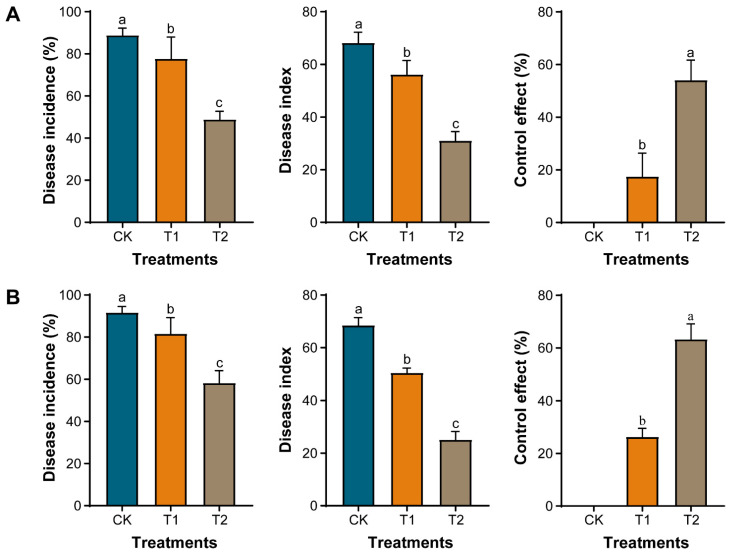
Effect of marigold crop rotation on the incidence of clubroot in Chinese cabbage under greenhouse and field conditions. Greenhouse (**A**) and field (**B**) conditions. Here, monocropping of Chinese cabbage (CK), Chinese cabbage seedlings were transplanted immediately after harvesting of the marigold crop (T1), and Chinese cabbage seedlings were transplanted with an empty period of 15 days after harvesting the marigold crop (T2). According to Duncan’s multiple range test (*p* < 0.05), different small letters on the error bars represent a significant difference among treatments.

Pot and field experiments were conducted under different conditions, to evaluate the effect of marigold crop rotation on the incidence of clubroot in Chinese cabbage. Data related to the effect of marigold crop rotation on disease incidence (%), disease index, and control effect (%) under greenhouse and field conditions are shown in Figure 3; Tables S1 and S2. In the greenhouse (Figure 3A) and field (Figure 3B and Figure S1) experiments, the control effect of marigold crop rotation (T1) was recorded at about 17.51% and 26.33%, respectively, compared with monocropping (CK). Whereas, when an empty period of 15 days was provided after the harvesting of the marigold crop and before transplanting the Chinese cabbage seedlings (T2), the control effect reached up to 54.13% and 63.35% under greenhouse (Figure 3A) and field (Figure 3B and Figure S1) conditions, respectively. The results showed that marigold crop rotation significantly controlled the incidence of clubroot in Chinese cabbage compared to monocropping. In contrast, the highest control effect was achieved when an empty period of 15 days was provided after harvesting the marigold crop, before transplanting the Chinese cabbage seedlings.

### 2.5. Marigold-Chinese Cabbage Crop Rotation Affects the Assembly, Diversity, and Structure of Rhizosphere Bacterial Communities

**Figure 4:**
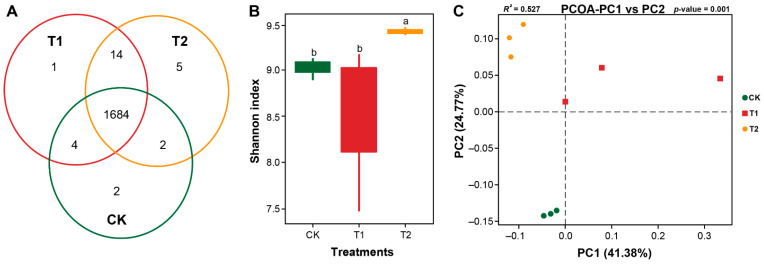
Diversity and structure of rhizosphere bacterial communities under marigold-Chinese cabbage crop rotation. Venn diagram showing the number of unique, shared, and common bacterial operational taxonomic units (**A**); Shannon diversity index for bacterial communities (**B**); and Principal coordinate analysis (PCoA) plots based on the Bray–Curtis dissimilarity matrix, showing the changes in the structure of bacterial communities (**C**). Different lowercase letters on each box represent the significant difference among treatments, according to a least significant difference test (LSD; *p* < 0.05). Monocropping of Chinese cabbage (CK), Chinese cabbage seedlings were transplanted immediately after harvesting the marigold crop (T1), and Chinese cabbage seedlings were transplanted with an empty period of 15 days after harvesting the marigold crop (T2).

A total of 720,225 raw reads (Avg; 80,025 reads/sample) ranging from 79,795 to 80,172 were obtained from all nine samples through high throughput amplicon sequencing of V3-V4 regions of 16S rRNA (Table 2). After quality control and chimera filtering, a total of 716,563 clean reads (Avg; 79,618 reads/sample) with an average length of 421 bps/sample were found (Table 2). The clean reads were then clustered into a total of 14,811 operational taxonomic units (OTUs) with an average of 1646 OTUs/sample at a ≥97% similarity level (Table 2). Furthermore, OTU analysis showed that a total of 1712 specific OTUs were recovered under different treatments (CK, T1, and T2), among which 1684 were found as common OTUs, whereas no significant difference was observed for common and unique OTUs (LSD, *p* < 0.05; Figure 4A). Within samples, an alpha diversity analysis for bacterial communities showed that values of the Shannon diversity index were significantly higher under T2 compared with CK and T1 (LSD, *p* > 0.05; Figure 4B), whereas no significant difference was observed between CK and T1 (LSD, *p* < 0.05; Figure 4B). Furthermore, we assessed the effect of marigold-Chinese cabbage crop rotation on the structure of rhizosphere bacterial communities under different treatments. A principal coordinate analysis (PCoA) based on a Bray–Curtis dissimilarity matrix showed a clear separation between CK, T1, and T2. The first two axes of PCoA showed a total 41.38 and 24.77% variation in the structure of rhizosphere bacterial communities (Figure 4C). The results of pairwise distances (PERMANOVA) between bacterial communities indicated that the structure of rhizosphere bacterial communities significantly changed under different treatments (*R^2^* = 0.527, *p* < 0.001).

### 2.6. Impact of Marigold-Chinese Cabbage Crop Rotation on Rhizosphere Bacterial Community Composition

**Figure 5:**
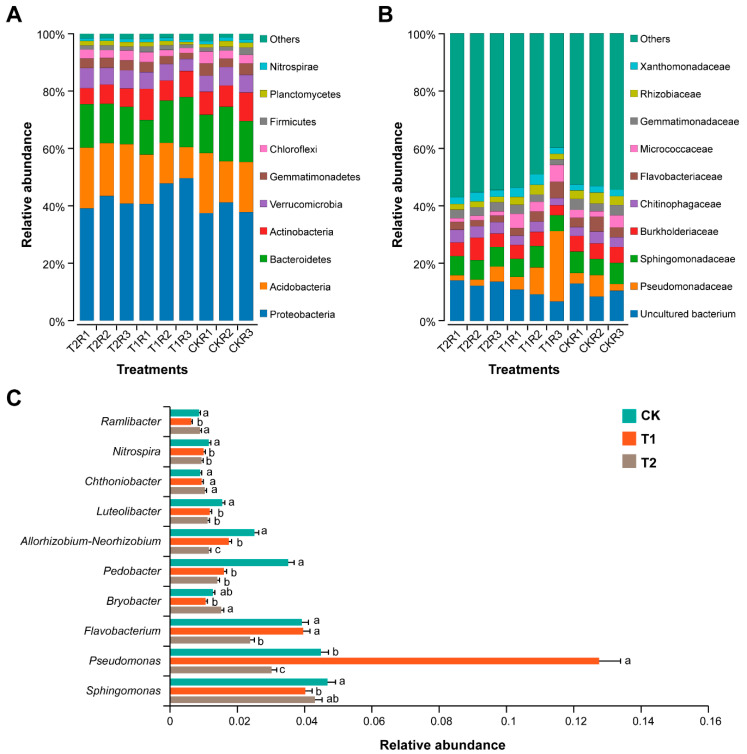
Distribution of most dominant bacterial communities at phylum, family, and genus levels under different treatments (± SEM; *n* = 3/treatment). Bar plots for the 10 most abundant dominant bacterial phyla (**A**), families (**B**), and genera (**C**). Different lowercase letters on the error bars represent the significant differences among treatments, according to a least significant difference test (LSD; *p* < 0.05). Monocropping of Chinese cabbage (CK), Chinese cabbage seedlings were transplanted immediately after harvesting the marigold crop (T1), and Chinese cabbage seedlings were transplanted with an empty period of 15 days after harvesting the marigold crop (T2).

Relative abundance (RA) bar plots, generated for the top-10 most abundant bacterial communities at phylum, family, and genus levels under different treatments (CK, T1, and T2), are shown in Figure 5. The phyla, such as Proteobacteria, Acidobacteria, Bacteroidetes, Actinobacteria, and Verrucomicrobia, were present in high RA and accounted for 87.77% of the total rhizosphere bacterial communities (Figure 5A and Table S3). Proteobacteria was present in high RA (46.04%) in the rhizosphere soil of T1 compared with CK and T2. The RA of Acidobacteria (20.09%) and Actinobacteria (6.20%) was increased and decreased, respectively, in the rhizosphere of T2 compared to CK and T1. Several phyla, such as Bacteroidetes, Verrucomicrobia, Gemmatimonadetes, Chloroflexi, Planctomycetes, Firmicutes, and Nitrospirae, were present as common in the rhizosphere soil, and no significant difference was observed in the RA of these across different treatments (LSD, *p* > 0.05). The family Pseudomonadaceae was highly dominant and significantly abundant in the rhizosphere soil of T1 compared to CK and T2 (LSD, *p* < 0.05; Figure 5B and Table S4). The RA of Flavobacteriaceae and Micrococcaceae was significantly decreased in the rhizosphere soil of T2 compared to CK and T1 (LSD, *p* < 0.05). In contrast, Sphingomonadaceae, Burkholderiaceae, Gemmatimonadaceae, and Xanthomonadaceae were present in the same RA in all rhizosphere soil samples, and no significant difference was observed among treatments (LSD, *p* > 0.05). At the genera level, the RA and taxonomic distribution patterns under different treatments became more obvious (Figure 5C and Table S5). *Pseudomonas* was significantly high RA in T1 compared to CK and T2 (LSD, *p* < 0.05). The RA of *Allorhizobium-Neorhizobium* decreased in the order CK > T1 > T2, and *Flavobacterium* was more present in high RA in CK and T1 than in T2 (LSD, *p* < 0.05). Whereas some bacterial genera, such as *Pedobacter*, *Luteolibacter*, and *Nitrospira*, had significantly high RA in CK compared to T1 and T2 (LSD, *p* < 0.05).

### 2.7. Correlation Analysis

**Figure 6:**
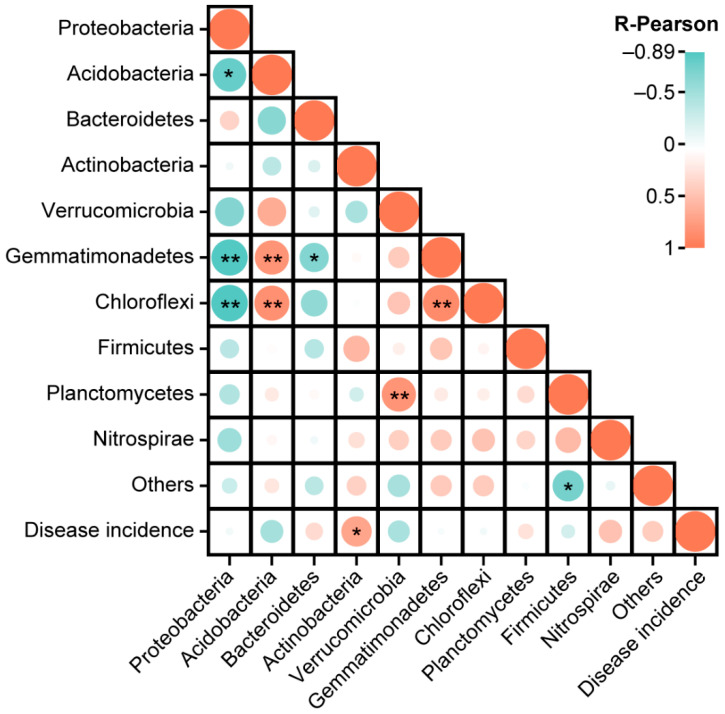
Pearson correlation analysis between the most abundant bacterial phyla and disease incidence. Pearson correlation coefficient (*p* < 0.05). Asterisks represents the significant differences at * *p* < 0.05 and ** *p* < 0.01.

A correlation analysis was performed at the phyla level using the Pearson correlation coefficient (PCC, *p* < 0.05), to further explore the impact of bacterial communities on disease occurrence. The results of PCC analysis showed that the phylum Actinobacteria was positively correlated (*p* < 0.05) with the disease incidence (Figure 6). This suggested that Actinobacteria enhance the population of clubroot pathogen *P. brassicae* and play an important role in disease acceleration.

### 2.8. Co-Occurrence Network Analysis of Rhizosphere Bacterial Communities

**Figure 7:**
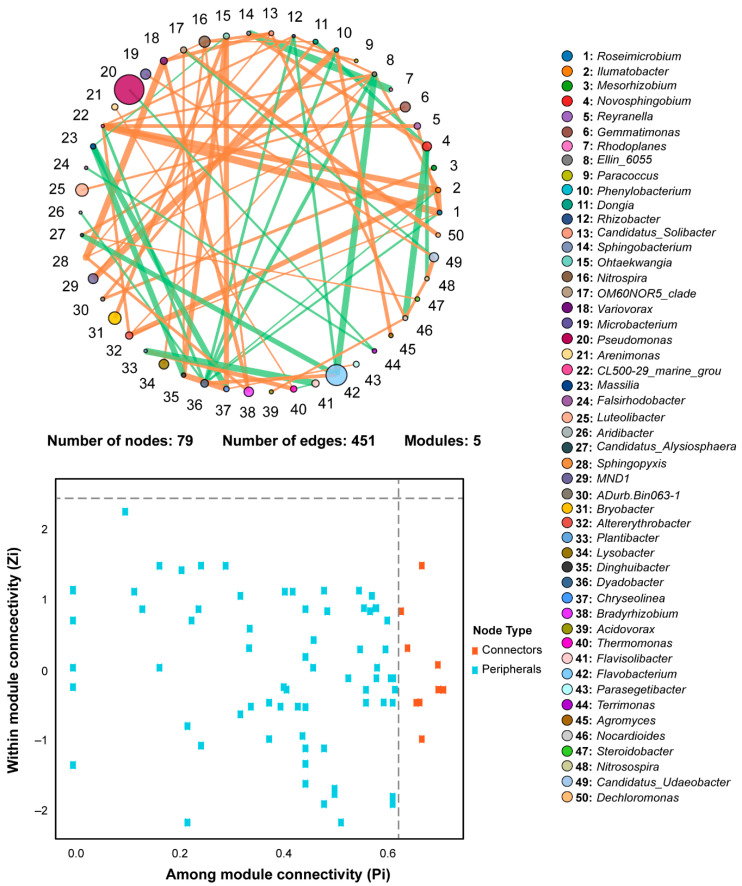
Co-occurrence network analysis of most abundant bacterial communities at genera level (correlation coefficient > 0.1 and *p* < 0.05).

The interaction between microorganisms in a complex microbial community is commonly studied by using co-occurrence network analysis. A microbial co-occurrence network was constructed for the top-50 bacterial genera, according to the abundance and variation of each species in each sample (Figure 7), and network properties are listed in Table S6. The microbial co-occurrence network was divided into 79 nodes, 451 edges, and 5 modules, showing that a complex microbial network existed among the rhizosphere bacterial communities. We observed a total of 902 degrees of connectivity among the rhizosphere bacterial communities under different treatments. A further co-occurrence analysis revealed a total of 172 strong negative correlations and 279 strong positive correlations among 50 bacterial genera. On average, the shortest path length between two nodes consisted of 2.418 edges, with a network diameter of 5 edges, whereas peripherals are opposite to connectors.

## 3. Discussion

Chinese cabbage (*Brassica rapa* subsp. *Pekinensis*) is an economically important vegetable crop that is widely cultivated all over China, and its production is seriously affected by the clubroot disease caused by the soilborne obligate biotroph parasite *Plasmodiophora brassicae* [31]. To date, integrated disease management approaches in chemical control, biological control, and resistant cultivars have been adopted, but they have limitations, and results have not been satisfactory [3]. Thus, it is of great importance to develop environmentally friendly IDM strategies in the form of crop rotation, to control this destructive pathogen, by breaking down its life cycle and enhancing the germination of resting spores. Over the past few decades, crop rotation has become a common way to enhance soil fertility, maintain soil biodiversity, and reduce pest and disease issues [32,33]. In this study, we assessed the impact of marigold–Chinese cabbage crop rotation on the incidence of clubroot in Chinese cabbage and soil bacterial communities through 16S amplicon sequencing in greenhouse and field experiments.

Marigold (*Tagetes erecta* L.) is reported to have antimicrobial properties and is also used for ornamental and pharmaceutical purposes [21]. Many previous studies have reported that intercropping, cover crop, and crop rotation with marigold significantly suppressed the incidence of soilborne disease, by enhancing the germination and death rate of resting spores and breaking the pathogen’s life cycle [20,25,34]. In this study, initially, we evaluated the effect of marigold seedlings on germination of *P. brassicae* resting spores in vitro. We observed that marigold root hairs significantly enhanced the germination of *P. brassicae* resting spores (Figure 1). Our results are similar to previous reports, where the primary life cycle of *P. brassicae* was examined in the root hairs and epidermal cells of non-cruciferous hosts [2], resulting in the death of *P. brassicae* spores due to the absence of a specific host for the secondary life cycle.

We assessed the impact of marigold root exudates, crude extract, powder, methanol, and sdH_2_O on the germination and death of *P. brassicae* resting spores and the incidence of clubroot on Chinese cabbage in vitro and in vivo. Our results confirmed that the germination and death rate of *P. brassicae* resting spores was significantly increased in marigold root exudates, crude extract, and powder compared to methanol and sdH_2_O (Figure 2). In contrast, the effect of marigold powder was more evident. The results are similar to previous studies, where marigold leaf extract had an allelopathic effect on *Chlorella vulgaris* cells [35]. The results of the greenhouse experiment revealed that the combined application of marigold powder and *P. brassicae* significantly suppressed the incidence of clubroot in Chinese cabbage, having a control effect of about 21.36% compared with the control (Table 1). However, the control effect was achieved up to 47.41% when *P. brassicae* spores were treated with marigold powder for 15 days in test tubes before the seedling was grown (Table 1). In greenhouse and field experiments, the control effect of marigold crop rotation was recorded at about 17.51% and 26.33%. In contrast, a control effect of about 54.13% and 63.35% was achieved when an empty period of 15 days was provided after harvesting the marigold crop, respectively. This may be due to the allelopathic effect of marigold and its ability to produce volatile thiophenes compounds, which caused the death of *P. brassicae* germinated spores. These results are similar to the previous reports that marigold crop rotation significantly reduces the damages caused by RKNs [36].

It was reported that marigold crop rotation under a greenhouse suppressed the incidence of *Ralstonia solanacearum* in tobacco plants [37]. Integrated treatment of marigold powder and *B. amyloliquefaciens* ZM9 significantly suppressed the incidence of tobacco bacterial wilt disease, by causing the death of *R. solanacearum* and enhancing the population of ZM9 in the rhizosphere of the tobacco plants [26]. Similarly, it was reported that marigold leaves caused *Alternaria solani* conidia death in in vitro conditions. Marigold–tomato intercropping suppressed the incidence of early tomato blight by reducing the *A. solani* conidial density around the tomato canopy, due to the allelopathic effect [20]. Many previous studies reported that marigolds can produce allelopathy compounds and successfully suppress plant-parasitic nematodes in many crops [22,24]. Thus, based on the above results, we speculated that by promoting the germination of *P. brassicae* in soil, the germinated zoospores cannot infect normally and die in the absence of a specific host, so as to reduce the primary source of infection.

In addition, we further investigated the effect of marigold–Chinese cabbage crop rotation on rhizosphere bacterial community composition and compared it with a monoculture cropping system. Microbes from distinctive phylogenetic lineages vary in their response to ecological changes [38]; thus, crop rotation may affect soil microbial community composition [33]. We noticed a significant shift in the rhizosphere bacterial community composition in the marigold-Chinese cabbage crop rotation system. The marigold-Chinese cabbage crop rotation and Chinese cabbage monocropping system did not differ in their unique and common OUTs, or alpha diversity indices. However, the results of PCoA, based on the Bray–Curtis dissimilarity matrix for rhizosphere bacterial communities, displayed a clear separation between different cropping systems, indicating that the rhizosphere bacterial community composition was significantly changed in the marigold-Chinese cabbage crop rotation and Chinese cabbage monocropping system. These results are in accordance with previous reports that marigold intercropping improves the alpha and beta diversity indices of soil microbial communities compared to a monocropping system [21].

Similarly, an integrated treatment of marigold and *B. amyloliquefaciens* ZM9 significantly suppressed the incidence of tobacco bacterial wilt disease, by improving the community composition of rhizosphere microbes [26]. This study suggested that Proteobacteria, Acidobacteria, Bacteroidetes, Actinobacteria, and Verrucomicrobia were the most dominant bacterial phyla in the rhizosphere soil of Chinese cabbage under different cropping systems. Our findings roughly correspond to the results of previous reports that agricultural soils, including the rhizosphere of Chinese cabbage, are significantly enriched in Proteobacteria and Bacteroidetes [31,39]. Acidobacteria was most abundant in the rhizosphere of the marigold-Chinese cabbage crop rotation cropping system; in contrast, the relative abundance of Actinobacteria was increased in the rhizosphere of the Chinese cabbage monocropping system. The significantly higher abundance of Proteobacteria in the marigold-Chinese cabbage crop rotation cropping system compared to the Chinese cabbage monocropping system indicated eutrophic soils, indicating that the soil health improved after intercropping, which is similar to previous reports [40,41].

## 4. Materials and Methods

### 4.1. Experimental Site and Plant Material

The pot experiments were performed in the greenhouse of the State Key Laboratory for Conservation and Utilization of Bio-Resources in Yunnan, Yunnan Agricultural University, Kunming (25°2′47.04″ N, 102°42′33.84″ E), China. The field experiments were performed in Dabai County, Panlong District, Kunming (25°2′47″ N, 102°42′33″ E), China, from May to November 2020. The average annual temperature and total rainfall per annum were recorded at about 15.1 °C and 1534 mm, respectively. Marigold variety Mengju No. 1 with a growth period of 100 days and Chinese cabbage variety 83-1, highly susceptible to clubroot pathogen, were provided by the Qingdao International Seed Co., LTD. (Qingdao, Shandong, China).

### 4.2. Preparation of Plasmodiophora Brassicae Spore Suspension

Root galls were collected from a field heavily infected with clubroot disease in Dabai County, Kunming, China, washed under tap water to remove soil, and stored at −20 °C until use. The root tissues were defrosted at room temperature (25–28 °C) for 5 days, and resting spores of *P. brassicae* were harvested, as previously described in [1], and identified as pathotype 4, according to the classification methodology of Jeong, et al. [42]. Briefly, 50 g of root gall was homogenized in (1:4, *w*/*v*) 200 mL of sterilized distilled water (sdH_2_O) in a mechanical blender and filtered through nylon cloth. The spore suspension was cleaned by washing 5 times with sdH_2_O at 5000 rpm for 7 min. Finally, the spore pellets were collected in the sediment and adjusted to a final concertation of 1 × 10^7^ spores/mL using a hemocytometer.

### 4.3. Assessment of the Effect of Marigold on the Germination of P. brassicae Resting Spores

To evaluate the effect of marigold on the germination of *P. brassicae* resting spores, marigold seedlings were grown hydroponically in a 10-mL test tube under dark (wrapped with black tape) at room temperature 28 ± 2 °C and inoculated with 100 μL/tube of *P. brassicae* spore suspension (1 × 10^7^ spores/mL) and sdH_2_O as a control (CK). Root hairs were collected after 24 h and 7 days of post-inoculation with *P. brassicae,* and germination of resting spores was visualized under a confocal microscope.

### 4.4. Marigold Root Exudates, Crude Extract, and Powder Preparation

Marigold root exudates, crude extract, and powder were prepared using the methodology of Zhang et al. [43] with some modifications.

#### 4.4.1. Preparation of Root Exudates

Marigold seedlings were grown hydroponically in a flask for one month, and Hoagland nutrient solution was applied three times a week to overcome the nutrient deficiencies. Then the marigold plants were taken out from the flask, roots were washed with sdH_2_O, placed in a beaker (wrapped with tin aluminum foil) containing 200 mL sdH_2_O, and the mouth of the beaker was air tightened with a sealing film. After 4 h of physiological activities under light, the liquid was collected, filtered, and extracted with twice the volume of ethyl acetate and concentrated on a rotary evaporator. The obtained product was dissolved in 2 mL of methanol, filtered through a 0.22-µm filter paper, adjusted to a final concentration of 0.046 mg/mL (stock solution), and stored at 4 °C for later use.

#### 4.4.2. Preparation of Marigold Tissues Crude Extract

The crude extract was prepared from 10 g of marigold tissues crushed in a blender. Briefly, 10 g of tissues were mixed in methanol (1:30, *w/v*), kept at room temperature for 24 h, and filtered through a cloth coffee filter. The extract was then concentrated on a rotary evaporator. Finally, a 0.0113 g of crude extract was obtained, dissolved in 2 mL of methanol (stock solution), and stored at 4 °C for future use.

#### 4.4.3. Preparation of Marigold Powder

For the Preparation of marigold powder, marigold plants (30 days old) were air-dried naturally in the shade and then crushed in a blender to make powder. Briefly, 1 g of marigold powder was mixed into 200 mL of sdH_2_O and boiled for 15 min. After that, the powder was filtered through a 0.22-µm filter paper and diluted with sdH_2_O to a constant volume of 100 mL, to prepare a stock solution, and stored at 4 °C for later use.

### 4.5. Analysis of Marigold Root Exudates, Crude Extract, and Powder on the Germination and Death of P. brassicae Resting Spores

To investigate the effect of marigold root exudates, crude extract, and powder on the germination and death of *P. brassicae* resting spores, Chinese cabbage seedlings were grown hydroponically in the dark (Figure S2). The seedlings were then inoculated with 100 μL/tube of *P. brassicae* spore suspension (1 × 10^7^ spores/mL), marigold root exudates (T1), crude extract (T2), and powder (T3), whereas control (CK) seedlings were treated with methanol (CK1) and sdH_2_O (CK2), respectively. The effects of different treatments on the death and germination of *P. brassicae* resting spores were observed 2 days after inoculation up to 16 days with an interval of 2 days. Briefly, a 100 μL suspension was obtained from each treatment and stained with 0.1% Evans solution for 7 h, as described by Hardin, et al. [44], and stained spores (200 spores/treatment; *n* = 3) were counted under a microscope. This assay was repeated thrice.

### 4.6. Greenhouse Experiments

#### 4.6.1. Evaluation the Effect of Marigold Powder on Clubroot

Chinese cabbage seedlings were grown hydroponically in the dark in test tubes, as mentioned above. The seedlings were then inoculated with 100 μL/tube of *P. brassicae* spore suspension (1 × 10^7^ spores/mL) and kept at room temperature 28 ± 2 °C. After 7 days post-treatment with *P. brassicae,* the seedlings were transplanted in pots containing double-sterilized soil and placed in the greenhouse under controlled conditions at 30 ± 2/ 20 ± 2 °C day/night temperature with a 14 h/10 h light/dark photoperiod. The experiment was performed under 3 conditions (Table 3). The experiment was repeated thrice with 9 pots per treatment, and 15 plants (3 pots)/treatment served as replicates.

#### 4.6.2. Investigation of Disease Incidence, Disease Index, and Control Effect

The disease incidence (Di), disease index (DI), and control effect (CE) were investigated after 30 days of transplantation using a 5-point disease grading scale, as described by Liu et al. [45]. The Di, DI, and CE were calculated using the formulas as follows: DI (%) = [Σ (number of diseased plants in each grade × disease grading scale)/(total number of plants under investigation × highest disease grading scale) × 100; Di = (number of diseased plants/total number of investigated plants) × 100; CE (%) = [(disease index of control-disease index of treatment)/disease index of control] × 100.

#### 4.6.3. Evaluation of the Effect of Marigold Crop Rotation on Clubroot Incidence in Chinese Cabbage

To further evaluate the effect of marigold crop rotation on mitigating clubroot incidence in Chinese cabbage, a pot experiment was conducted under controlled conditions in a greenhouse, as described above. Marigold and Chinese cabbage seedlings were grown hydroponically and in a polystyrene tray 4 and 3 weeks prior to use, respectively, according to the methodology of Li et al. [46]. The seedlings were transplanted in pots filled with diseased soil collected from a field (heavily infected with clubroot pathogen *P. brassicae*) at Dabai County, Kunming, China. The Di, DI, and C.E were calculated after 50 days of Chinese cabbage seedling transplantation, as previously described by Liu et al. [45]. The experiment was performed under 3 conditions (Table 4) and repeated thrice with a total of 45 plants per treatment (15 plants/replication).

### 4.7. Field Experiment

A field experiment (marigold crop rotation) was conducted at Dabai County, Kunming City, from June to November 2020 in the monocropping soil of Chinese cabbage heavily infected with clubroot pathogen *P. brassicae*. Chinese cabbage crops had been continuously grown in the field for the previous 10 years, and in the last cropping season, the Di and DI were recorded at 100% and 85.31%, respectively. The experiment was performed under 3 conditions (Table 5). Marigold and Chinese cabbage seedlings were transplanted in plots (1.2 × 1 m) on ridges, and a P × P distance was about 20 cm. The experiment was conducted under a randomized complete block design and repeated thrice with a total of 3 plots/treatment, and 20 plants/treatments served as replicates. The Di, DI, and CE were calculated after the harvesting of Chinese cabbage crop, as mentioned above [45].

### 4.8. Soil Samples Collection and DNA Extraction

In addition, a high-throughput sequencing tool was used to explore the effect of marigold-Chinese cabbage crop rotation on rhizosphere bacterial diversity and community composition. The soil samples were collected from a field experiment in replicates (minimum 3 biological replication/treatment) from each treatment, according to the methodology of Ahmed et al. [47]. Briefly, 10 plants/plot were uprooted, bulk soil was removed from the roots by gently shaking the plants, and soil particles adhered to roots were collected as rhizosphere soil samples for rhizosphere bacterial diversity analysis. Total soil DNA was extracted from 0.5 g of soil/sample using a PowerSoil^®^ DNA extraction Kit (MO BIO Laboratories, Carlsbad, CA, USA), by following the manufacturer’s instructions, and extracted DNA quality was quantified at OD_260/280 nm_ 1.7–1.9 using a NanoDrop spectrophotometer (ND2000, Thermo Scientific Waltham, MA, USA). The extracted DNA was stored at −20 °C for PCR amplification and library construction.

### 4.9. High Throughput Amplicon Sequencing and Analysis of Rhizosphere Bacterial Diversity

The V3-V4 variable region of 16s rRNA was amplified using primer pair 343F (5′-TACGGRAGGCAGCAG-3′) and 798R (5′-AGGGTATCTATCCT-3′) [47], and PCR products were sequenced on an Illumina MiSeq platform at Tsingke Biotechnology Co., LTD. (Beijing, China). Raw data collected from Illumina sequence in FASTQ format were then quality controlled at a 20% cutoff level using Trimmomatic software V.0.33 [48], and chimeras were removed with UCHIME V.8.1 [49]. The cleans reads were then processed on the UPARSE pipeline, to cluster into operational taxonomic units (OTUs) at a 3% dissimilarity level [50] and blasted against the Ribosomal Database Project (RDP) classifier in the SLIVA database (http://www.arb-silva.de accessed on 17 July 2022) of bacteria for taxonomic annotation at a 70% threshold level [51].

### 4.10. Bioinformatics Analysis

Alpha diversity indices (Chao 1, Shannon, etc.) for bacterial communities were calculated using QIIME v.1.9.1. Principal Coordinate Analysis (PCoA) based on the Bray-Curtis dissimilarity matrix, to visualize the changes in bacterial community structure. Permutational multivariate analysis of variance (PERMANOVA) was performed using the adonis() function from the vegan package in R to confirm the changes in the bacterial communities [52]. Relative abundance bar plots at the phylum, family, and genus levels were generated using the barplot() function in the “ggplot2” package in R v4.2.1. A correlation analysis was performed between disease incidence and the most abundant bacterial phyla using Pearson correlation coefficient (PCC, *p* < 0.05) in the ggcor package “ggplot2” and visualized through a heatmap. Spearman correlation was used to construct a microbial co-occurrence network, according to the abundance and variation of each species in each sample (Spearman, default method), with rank correlation analysis and screening correlation coefficient > 0.1 and *p* < 0.05. The nodes were divided into four categories according to Zi and Pi values, as follows: Peripheral nodes (Zi ≤ 2.5, Pi ≤ 0.62), Connectors (Zi ≤ 2.5, Pi > 0.62), Module hubs (Zi > 2.5, Pi ≤ 0.62), and Network hubs (Zi > 2.5, Pi > 0.62). Data were statistically analyzed using analysis of variance (ANOVA) in Microsoft excel 2019, and means were compared using least significant difference (LSD) and Duncan’s multiple range test at *p <* 0.05 in IBM SPSS Statistics V.24.0.

## 5. Conclusions

The results of our study suggest that marigold can be used as a trap plant for the prevention and control of clubroot in Chinese cabbage. In vitro experiments confirmed that marigold powder, crude extract, and root exudates significantly promoted the germination and death of *P. brassicae* resting spores. Greenhouse and field experiments confirmed that marigold–Chinese cabbage crop rotation significantly improved the bacterial community composition and suppressed the clubroot incidence in Chinese cabbage when an empty period of 15 days was provided before the transplanting of Chinese cabbage seedlings after harvesting the marigold crop. This also provides a new idea for sustainable agriculture and for the successful prevention and control of cruciferous vegetables clubroot disease. However, the impact of marigold–Chinese cabbage crop rotation on the soil physicochemical properties, enzymatic activity, functional potential of soil microbial communities, and fungal diversity is still unclear and needs further study.

## Figures and Tables

**Table 1:** Effect of marigold powder on clubroot in Chinese cabbage.

Treatment	Disease Incidence (%)	Disease Index	Control Effect (%)
CK	86.67 ± 6.67 a	63.38 ± 1.80 a	-----
T1	71.11 ± 7.70 b	49.84 ± 1.46 b	21.36 ± 1.37 b
T2	46.67 ± 6.67 c	33.33 ± 2.86 c	47.41 ± 4.40 a

CK; sdH_2_O (100 μL/tube) + *P. brassicae* spore suspension (100 μL/tube) + Chinese cabbage seedling, T1; marigold powder (100 μL/tube) + *P. brassicae* spore suspension (100 μL/tube) + Chinese cabbage seedling, T2; marigold powder (100 μL/tube) + *P. brassicae* spore suspension (100 μL/tube) treated for 15 days + Chinese cabbage seedling. Different lowercase letters within the column show the significant differences among treatments according to Duncan’s multiple range test at *p* < 0.05. Note: In T2, initially, *P. brassicae* resting spores were treated in test tubes with marigold powder for 15 days before growing the Chinese cabbage seedlings.

**Table 2:** High throughput sequencing data through amplification of the V3-V4 variable region of 16S rRNA.

Sample ID	Raw Reads	Clean Reads	Average Length (bp)	Number of OTUs
T2-R1	79,899	79,465	421	1658
T2-R2	79,901	79,507	421	1669
T2-R3	79,795	79,399	421	1683
T1-R1	80,037	79,623	420	1676
T1-R2	80,172	79,762	421	1656
T1-R3	80,138	79,741	421	1620
CK-R1	80,116	79,701	420	1648
CK-R2	80,118	79,723	421	1556
CK-R3	80,049	79,642	420	1645
Total	720,225	716,563	-------	14,811
Average	80,025	79,618	421	1646

Monocropping of Chinese cabbage (CK), Chinese cabbage seedlings were transplanted immediately after harvesting the marigold crop (T1), and Chinese cabbage seedlings were transplanted with an empty period of 15 days after harvesting the marigold crop (T2). R1, R2, and R3 represent the number of samples per treatment.

**Table 3:** Experimental conditions.

Treatments	Conditions
CK	sdH_2_O (100 μL/tube) + *P. brassicae* spore suspension (100 μL/tube) + Chinese cabbage seedling
T1	marigold powder (100 μL/tube) + *P. brassicae* spore suspension (100 μL/tube) + Chinese cabbage seedling
T2	marigold powder (100 μL/tube) + *P. brassicae* spore suspension (100 μL/tube) treated for 15 days + Chinese cabbage seedling

sdH_2_O; sterilized distilled water. In T2, initially, *P. brassicae* resting spores were treated in test tubes with marigold powder for 15 days before growing the Chinese cabbage seedlings.

**Table 4:** Experimental conditions.

Treatments	Conditions
CK1	Monocropping of Chinese cabbage
T1	Chinese cabbage seedlings were transplanted immediately after harvesting the marigold crop
T2	Chinese cabbage seedlings were transplanted 15 days after harvesting the marigold crop

In T2, we provided an empty period of 15 days after harvesting the marigold crop and before the transplantation of Chinese cabbage seedlings.

**Table 5:** Experimental conditions.

Treatments	Conditions
CK	Monocropping of Chinese cabbage
T1	Crop rotation (marigold crop + Chinese cabbage crop)
T2	Crop rotation (marigold crop + 15 days empty period + Chinese cabbage crop)

In treatment (T1), Chinese cabbage seedlings were immediately transferred after harvesting the marigold crop. Whereas in treatment (T2), an empty period of 15 days was provided after harvesting the marigold crop and before the transplantation of Chinese cabbage seedlings.

## Data Availability

All raw data related to 16S amplicon sequencing have been submitted to the public database NCBI and are available as a Sequence Read Archive (SRA) under BioProject No. PRJNA859131.

## Supplementary Materials

The following supporting information can be downloaded at: https://www.mdpi.com/article/10.3390/plants11172295/s1. Figure S1; Effect of marigold crop rotation on the incidence of clubroot in Chinese cabbage under field conditions. Figure S2; Chinese cabbage seedlings were grown hydroponically in the dark to investigate the effect of marigold root exudates, crude extract, and powder on the germination and death of *P. brassicae* resting spores. Table S1; Effects of inoculation of marigold on the disease incidence, disease index, and control effect of cabbage clubroot in the greenhouse. Table S2; Effects of inoculation of marigold on the disease incidence, disease index, and control effect of cabbage clubroot in the field. Table S3; Relative abundance of the top-10 dominant bacterial phyla in rhizosphere soil under different experimental conditions (±SEM, *n* = 3). Table S4; Relative abundance of the top-10 dominant bacterial family in rhizosphere soil under different experimental conditions (±SEM, *n* = 3). Table S5; Relative abundance of the top-15 dominant bacterial genera in rhizosphere soil under different experimental conditions (±SEM, *n* = 3). Table S6; Characteristics of the co-occurrence network.
