# Supplementary material for: Crop Rotation with Marigold Promotes Soil Bacterial Structure to Assist in Mitigating Clubroot Incidence in Chinese Cabbage
Source: Plants (Basel). 2022 Sep 2;11(17):2295. doi: 10.3390/plants11172295 (PMC9460896; doi:10.3390/plants11172295)
Supplement: Supplementary file 1 [file plants-11-02295-s001.zip › plants-1846218-supplementary.pdf]

Crop rotation with marigold promotes soil bacterial structure to assist in mitigating clubroot incidence in Chinese cabbage

Jinhao Zhang [1,2,†], Waqar Ahmed[1,2,†], Xinghai Zhou[1,2,†], Bo Yao[1,2], Zulei He [1,2], Yue Qiu[1,2], Fangjun Wei[1,2], Yilu He[1,2], Lanfang Wei [1,3]* and Guanghai Ji [1,2]*

[1]State Key Laboratory for Conservation and Utilization of Bio-Resources in Yunnan, Yunnan Agricultural University, Kunming 650201, Yunnan, China

[2]Key Laboratory of Agro-Biodiversity and Pest Management of Ministry of Education, Yunnan Agricultural University, Kunming 650201, Yunnan, China

[3]Agricultural Foundation Experiment Teaching Center, Yunnan Agricultural University, Kunming 650201, Yunnan, China

†These authors contributed equally to this work.

***Correspondence:**

Guanghai Ji

jghai001@163.com

Lanfang Wei

wlfang2000@aliyun.com

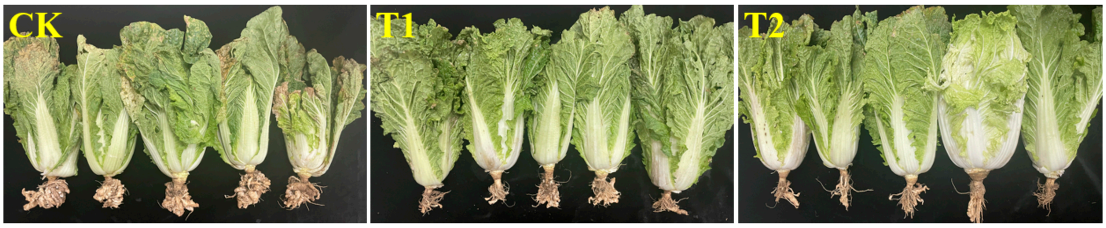

Figure S1; Effect of marigold crop rotation on the incidence of clubroot in Chinese cabbage under field conditions. **Here;** Monocropping of Chinese cabbage **(CK)**, Chinese cabbage seedlings were transplanted immediately after harvesting of the marigold crop **(T1)**, and Chinese cabbage seedlings were transplanted with an empty period of 15 days after harvesting of the marigold crop **(T2)**.

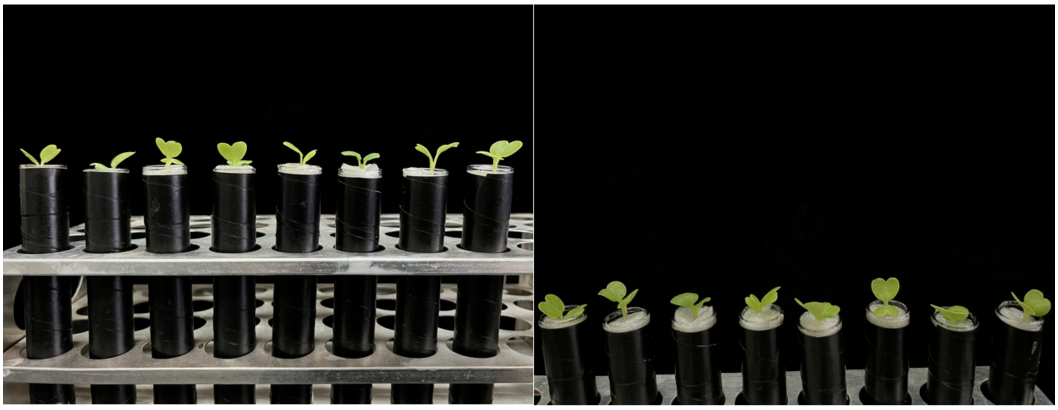

Figure S2; Chinese cabbage seedlings were grown hydroponically in the dark to investigate the effect of marigold root exudates, crude extract, and powder on the germination and death of *P. brassicae* resting spores.

Table S1; Effects of inoculation of marigold on the disease incidence, disease index, and control effect of cabbage clubroot in the greenhouse.

Treatment	Disease incidence (%)	Disease index	Control effect (%)
CK	88.89±3.85a	68.25±3.96a	-----
T1	77.78±10.18b	56.19±5.30b	17.51±8.82b
T2	48.89±3.85c	31.11±3.35c	54.13±7.56a

Note: Monocropping of Chinese cabbage **(CK)**, Chinese cabbage seedlings were transplanted immediately after harvesting of marigold crop **(T1)**, and Chinese cabbage seedlings were transplanted with an empty period of 15 days after harvesting of marigold crop **(T2)**. Significance differences among treatments are shown by different small letters within a column according to Duncan's multiple range test at $p < 0.05$.

Table S2; Effects of inoculation of marigold on the disease incidence, disease index, and control effect of cabbage clubroot in the field.

Treatment	Disease incidence (%)	Disease index	Control effect (%)
CK	91.67±2.89a	68.57±2.85a	-----
T1	81.67±7.64b	50.47±1.80b	26.33±3.22b
T2	58.33±5.77c	25.14±3.06c	63.35±5.80a

Note: Monocropping of Chinese cabbage **(CK)**, Chinese cabbage seedlings were transplanted immediately after harvesting of marigold crop **(T1)**, and Chinese cabbage seedlings were transplanted with an empty period of 15 days after harvesting of marigold crop **(T2)**. According to Duncan's multiple range test different small letters within a column shows significance differences among treatments at $p < 0.05$.

Table S3; Relative abundance of the top 10 dominant bacterial phyla in rhizosphere soil under different experimental conditions (±SEM, n=3).

Phylum	CK	T1	T2
Proteobacteria	38.80±2.07bc	46.04±4.74a	41.11±2.20b
Acidobacteria	17.60±3.31ab	14.06±3.18b	20.09±1.49a
Bacteroidetes	15.54±3.03a	14.73±2.71a	13.97±1.06a
Actinobacteria	8.45±1.36a	8.96±1.94a	6.20±0.57b
Verrucomicrobia	6.04±0.44a	5.27±0.90a	6.41±0.60a
Gemmatimonadetes	3.75±0.72a	2.83±0.69a	3.46±0.12a
Chloroflexi	3.31±0.74a	2.41±0.94a	3.07±0.37a
Firmicutes	1.80±0.68a	1.62±0.36a	1.49±0.17a
Planctomycetes	1.60±0.43a	1.30±0.37ab	1.59±0.02a
Nitrospirae	1.15±0.06a	1.00±0.17a	0.94±0.09a
Others	1.94±0.61a	1.78±0.33a	1.67±0.17a

Here; Monocropping of Chinese cabbage **(CK)**, Chinese cabbage seedlings were transplanted immediately after harvesting of marigold crop **(T1)**, and Chinese cabbage seedlings were transplanted with an empty period of 15 days after harvesting of marigold crop **(T2)**. Different lowercase letters within a row represents the significance differences among treatments according to a least significant difference test (LSD; $p < 0.05$).

Table S4; Relative abundance of the top 10 dominant bacterial families in rhizosphere soil under different experimental conditions (±SEM, n=3).

**Family**	**CK**	**T1**	**T2**
Pseudomonadaceae	4.49±2.65b	12.75±10.44a	3.02±1.92c
Sphingomonadaceae	6.80±1.03a	6.42±0.96a	6.77±0.07a
Burkholderiaceae	5.45±0.07a	4.37±0.82a	5.73±1.81a
Chitinophagaceae	3.56±0.56ab	3.11±0.56b	4.12±0.28a
Flavobacteriaceae	3.92±1.03a	3.96±1.54a	2.38±0.32b
Micrococcaceae	2.95±1.14b	4.72±1.27a	1.47±0.15c
Gemmatimonadaceae	3.37±0.64a	2.53±0.59a	3.00±0.12a
Rhizobiaceae	3.28±0.46a	2.69±0.75ab	1.99±0.07b
Xanthomonadaceae	2.20±0.13a	3.00±0.75a	2.61±0.51a
Uncultured bacterium	10.66±2.27ab	8.97±2.06b	13.34±0.96a
Others	53.34±0.91a	47.47±7.11a	55.57±1.29a

Here; Monocropping of Chinese cabbage **(CK)**, Chinese cabbage seedlings were transplanted immediately after harvesting of marigold crop **(T1)**, and Chinese cabbage seedlings were transplanted with an empty period of 15 days after harvesting of marigold crop **(T2)**. Significance differences among treatments are shown by different small letters within a row according to a least significant difference test (LSD; $p < 0.05$).

Table S5; Relative abundance of the top 15 dominant bacterial genera in rhizosphere soil under different experimental conditions (±SEM, n=3).

**Genus**	**CK**	**T1**	**T2**
Pseudomonas	0.045±0.027b	0.127± 0.104a	0.030±0.019c
Sphingomonas	0.047±0.006a	0.040±0.007a	0.043±0.002ab
Flavobacterium	0.039±0.010a	0.039±0.015a	0.024±0.003b
Pedobacter	0.035±0.006a	0.016±0.006b	0.014±0.003b
Allorhizobium-Neorhizobium	0.025±0.004a	0.017±0.005b	0.012±0.001c
Bryobacter	0.013±0.001ab	0.010±0.002b	0.015±0.002a
Luteolibacter	0.016±0.002a	0.012±0.003b	0.011±0.001b
RB41	0.014±0.003a	0.011±0.003a	0.011±0.002b
Nitrospira	0.012±0.001a	0.010±0.001b	0.010±0.001b
Chthoniobacter	0.009±0.001a	0.009±0.001a	0.010±0.001a
MND1	0.008±0.001b	0.007±0.004b	0.010±0.000a
Ramlibacter	0.009±0.001a	0.006±0.002b	0.009±0.001a
Novosphingobium	0.004±0.001b	0.009±0.002a	0.008±0.000a
JGI_0001001-H03	0.008±0.001a	0.005±0.001a	0.009±0.001a
Delftia	0.000±0.000b	0.0008±0.0005b	0.009±0.015a

Here; Monocropping of Chinese cabbage **(CK)**, Chinese cabbage seedlings were transplanted immediately after harvesting of marigold crop **(T1)**, and Chinese cabbage seedlings were transplanted with an empty period of 15 days after harvesting of marigold crop **(T2)**. Different lowercase letters within a row represents the significance differences among treatments according to a least significant difference test (LSD; $p < 0.05$).

Table S6; Characteristics of the co-occurrence network.

Network properties	Value
Number of nodes	79
Number of edges	451
Modularity	0.442
Number of communities	6
Network diameter	5
Network Density	0.146
Average shortest path length	2.418
Average clustering coefficient	0.503
